# Loss of large tumor suppressor 1 promotes growth and metastasis of gastric cancer cells through upregulation of the YAP signaling

**DOI:** 10.18632/oncotarget.7568

**Published:** 2016-02-22

**Authors:** Jing Zhang, Ge Wang, Shao-Jun Chu, Jin-Shui Zhu, Rui Zhang, Wen-Wen Lu, Li-Qiong Xia, Yun-Min Lu, Wei Da, Qun Sun

**Affiliations:** ^1^ Department of Gastroenterology, Shanghai Jiao Tong University Affiliated Shanghai Sixth People's Hospital, Shanghai 200233, China; ^2^ Department of Gerontology, Shanghai Jiao Tong University Affiliated Shanghai Sixth People's Hospital, Shanghai 200233, China

**Keywords:** large tumor suppressor 1, YAP, metastasis, prognosis, gastric cancer

## Abstract

Accumulating evidence shows that large tumor suppressor 1 (LATS1) as a novel resident governor of cellular homeostasis is implicated in multiple tumorigenic properties including cell growth, apoptosis and metastasis. However, the contribution of LATS1 to gastric carcinoma (GC) remains unclear. The correlation of LATS1 expression with clinicopathologic characteristics, GC prognosis and recurrence was analyzed by immunohistochemistry, Univariate and Kaplan-Meier analysis. Functional experiments were performed to investigate biological behaviors of GC cells and underlying molecular mechanisms. Tumor growth and metastasis was assessed *in vivo* using orthotopic implantation GC models in severe combined immune deficiency (SCID) mice. Consequently, decreased LATS1 expression was significantly associated with the lymph node metastasis, poor prognosis and recurrence. Ectopic expression of LATS1 decreased GC cell proliferation and invasion *in vitro* and inhibited tumor growth and liver metastasis *in vivo*, but depletion of LATS1 expression restored the invasive phenotype. Further observation indicated that YAP pathway was required for LATS1-induced inhibition of cell growth and invasion, and LATS1 restrained nuclear transfer of YAP, downregulated YAP, PCNA, CTGF, MMP-2, MMP-9, Bcl-2 and CyclinD1 expression and upregulated p-YAP and Bax expression. Our findings suggest that LATS1 is a potential candidate tumor suppressor and inhibits the growth and metastasis of GC cells via downregulation of the YAP signaling.

## INTRODUCTION

Although worldwide incidence of gastric cancer (GC) is gradually decreasing, it is still one of the leading causes of cancer death and the most common gastrointestinal malignancy in East Asia threatening the public health [1, 2]. Recent decades have witnessed the efforts in improving the treatment of GC via surgical therapies and adjuvant chemotherapy, but the prognosis of GC in advanced stage remains unsatisfactory [3]. Therefore, a comprehensive understanding of the molecular mechanisms associated with tumor development and progression is essential for early diagnosis and treatment of GC [4].

Large tumor suppressor (LATS) gene family has aroused widespread concern in recent years due to its potential as novel resident governor of cellular homeostasis as well as p53 or Ras family [5]. The representative members of LATS family, such as LATS1 and LATS2, a kind of Ser/Thr kinase originally isolated from Drosophila [6, 7], are implicated in many crucial life processes including cell proliferation, cell apoptosis and cell migration [8] and surveillant behaviors in transcriptional regulation and maintenance of genetic stability [9]. Aberrant expression or gene mutation of LATS1 contributes to malignant transformation and histological progression in cervical squamous cell carcinoma (CSCC) [10], breast cancer [11], hepatocellular carcinoma (HCC) [12] and astrocytoma [13]. Overexpression of LATS1 significantly suppresses human renal carcinoma cell growth and tumorigenicity *in vivo* [14]. Gene mutations in key protein domains [15] and methylation in promoter region [13] frequently occur in human stomach adenocarcinoma and astrocytoma tissues and eradicates normal function of LATS1 leading to the production of neoplasm. Hence, detection of gene mutations in LAST1 may be a useful tool for cancer diagnosis and prognostic indicator [16].

The Hippo pathway known as regulating the balance between cell proliferation and apoptosis consists of Mst1/2, SAV1, Lats1/2, Mob and yes-associated protein (YAP) and participates in inhibition of proliferation as well as organ size control [17]. As the nuclear effector of Hippo pathway, YAP originally identified from Drosophila Yorkie (yki) is shown to be a potent oncoprotein [18], and its inactivation results in the restoration of cell contact inhibition and growth control [19]. YAP is overexpressed in a variety of cancers, such as HCC [20], non-small cell lung cancer (NSCLC) [21], breast cancer [22], melanoma [23], hedgehog-associated medulloblastomas [24], colonic adenocarcinoma, ovarian serous cystadenocarcinoma [25] and lung adenocarcinoma [26]. Loss of YAP is negatively associated with estrogen and progesterone receptors in invasive breast carcinomas [27]. Disruption of LATS1 by heat shock protein 90 inhibitors promotes tumor proliferation, metastasis, and angiogenesis [28], indicating that LATS1 may act a pivotal role in the formation and progression of malignant tumors.

It is reported that LATS1 contributes to good prognosis and negatively regulates YAP oncoprotein in NSCLC [29], but downregulation of YAP decreases the expression of LATS1 in HCC cells [30]. The relationship between LATS1 and YAP expression in regulating gastric tumorigenesis is further explored. Our previous studies have proved that the expression of LATS1 is downregulated and negatively associates with YAP in GC tissues [31], whereas silencing of YAP reduces the growth and invasion in GC cells [32]. However, little is known concerning the function of LATS1 and its molecular regulatory mechanisms in GC cells. In the present study, we hypothesized that decreased expression of LATS1 was associated with tumor metastasis and the poor prognosis and recurrence in GC patients and overexpression of LATS1 suppressed growth and metastasis in GC cells through inhibition of the YAP signaling.

## RESULTS

### The expression of LATS1 in GC tissues and cell lines

**Figure 1:**
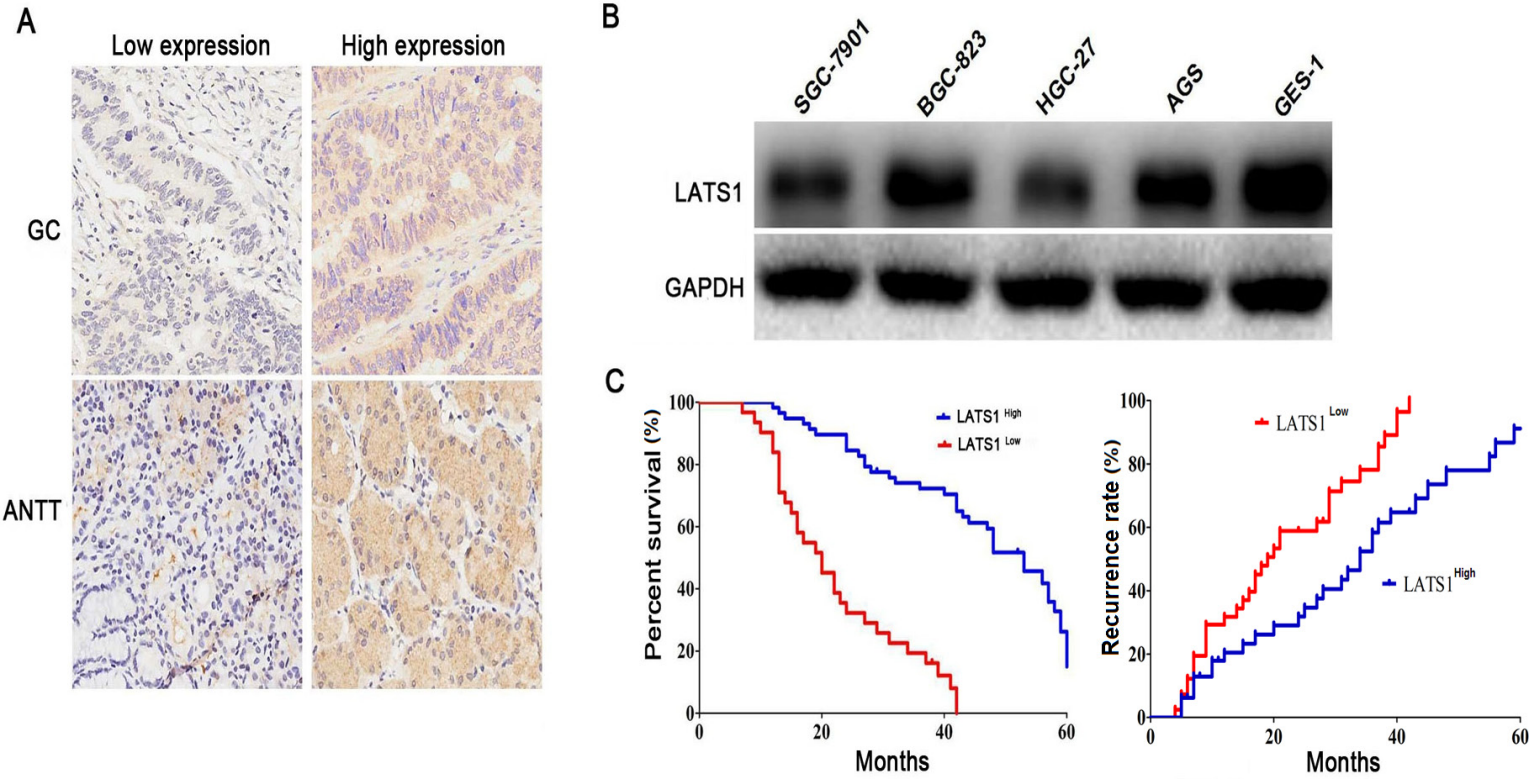
LAST1 was lowly expressed in GC tissues and cell lines (**A**) Differential protein expression levels (low or high) of LATS1 were examined in GC tissues and ANTT by IHC staining (magnification, 200×). (**B**) Western blotting analysis of LATS1 expression in GC cell lines. (**C**) Low expression of LATS1 was markedly correlated with the overall survival and recurrence free survival of the GC patients. These data were analyzed using Kaplan-Meier survival analysis between the GC patients with low LATS1 expression and high LATS1 expression according to the immunostaining scores.

Previous studies have shown that LATS1 expression is downregulated in malignant tumors, including CSCC [10], breast cancer [11] and HCC [12]. To examine the expression of LATS1 in GC tissues, we detected the expression level of LATS1 in 89 cases of GC patients with paired adjacent non-tumor tissue (ANTT) by IHC. The results showed that the differential protein expression levels of LATS1 were identified in GC tissues and ANTT (Figure 1A), and LATS1 expression was markedly decreased in GC tissues compared with that in ANTT (*P* < 0.001, Table 1). To evaluate whether GC cells presented decreased LATS1 level, we investigated the LATS1 expression in GC cell lines using Western blotting (Figure 1B), and found that the LATS1 protein expression was significantly downregulated in GC cell lines, especially in invasive SGC-7901 and HGC-27 ones, compared with the human gastric epithelial cells GES-1.

**Table 1:** The expression of LATS1 in human GC tissues

Target	Group	*N*	LATS1 expression				Positive rate (%)	χ^2^	*P*
			negative	low	intermediate	strong			
LATS1	GC	89	23	35	14	17	74.16		
	ANTT	89	6	20	27	36	93.26	22.533	< 0.001

GC: gastric cancer; ANTT: adjacent non-tumor tissues

### Association of LATS1 expression with clinicopathologic features, prognosis and recurrence in GC patients

The low expression of LATS1 in GC tissues inspired us to further analyze the clinical relevance of LATS1 expression with the progression, prognosis and recurrence in GC patients. The association of LATS1 expression with clinicopathologic characteristics was assessed in Table 2. Decreased expression of LATS1 was associated with the lymph node metastasis (*P* = 0.012). However, no correlations were found between LATS1 expression and other clinical features, including age, gender, tumor size, pathological staging and T/N classification (*P* > 0.05). Kaplan-Meier analysis using the log-rank test showed that GC patients with low LATS1 expression had shorter median survival time of 27.3 months and median recurrent time of 20.6 months, while those with high LATS1 expression had median survival time of 45.6 months and median recurrent time of 34.7 months (*P* = 0.0013 and *P* = 0.0029; Figure 1C). Multivariate analysis showed that, in addition to the lymph node metastasis, LATS1 expression might act as an independent prognostic factor for overall survival (OS) (*P* = 0.017, Table 3) and recurrence-free survival (RFS) (*P* = 0.026, Table 4) of GC patients.

**Table 2:** The correlation of LATS1 protein expression with clinicopathologic features in GC patients

Clinicopathologic features	Cases (*n*)	LATS1 Expression		*P*
		Low	High	
**Age**				
≥ 60	29	18	11	
< 60	60	40	20	0.871
**Gender**				
Female	20	15	5	
Male	69	43	26	0.297
**Tumor size (cm)**				
< 3.5	17	9	8	
≥ 3.5	72	49	23	0.242
**Pathological stage**				
I/II	32	21	11	
III/IV	57	37	20	0.946
**T classification**				
T1/T2	11	8	3	
T3/T4	78	50	28	0.576
**N classification**				
N0/N1	39	26	13	
N2/N3	50	32	18	0.794
**Lymph node metastasis**				
Negative	23	10	13	
Positive	66	48	18	0.012

**Table 3:** Summary of univariate and multivariate Cox regression analysis of overall survival duration

Parameter	Univariate *P*	Multivariate analysis		
		*P*	HR	95%CI
Age (≥ 60 vs. < 60 years)	0.112	NA		
Gender (Male vs. Female)	0.282	NA		
Tumor size (≥ 3.5 vs. < 3.5 cm)	0.313	NA		
Pathological stage (I/II vs. III/IV)	0.012	NS	1.872	0.872–1.478
T classification (T1/T2 vs. T3/T4)	0.0087	NS	1.367	1.257–2.977
N classification (N0/N1 vs. N2/N3)	0.023	NS	2.091	1.989–2.657
Lymph node metastasis (Positive vs. Negative)	0.0027	0.029	1.327	1.251–2.077
LATS1 expression (High vs. low)	0.0013	0.017	0.917	0.842–1.538

NA: not analyzed; NS: not significant.

**Table 4:** Summary of univariate and multivariate Cox regression analysis of recurrence rate duration

Parameter	Univariate *P*	Multivariate analysis		
		*P*	HR	95%CI
Age (≥ 60 vs. < 60 years)	0.371	NA		
Gender (Male vs. Female)	0.147	NA		
Tumor size (≥ 3.5 vs. < 3.5 cm)	0.269	NA		
Pathological stage (I/II vs. III/IV)	0.024	NS	1.297	1.031–1.938
T classification (T1/T2 vs. T3/T4)	0.012	NS	1.677	1.512–2.822
N classification (N0/N1 vs. N2/N3)	0.039	NS	2.513	1.096–1.872
Lymph node metastasis (Positive vs. Negative)	0.0048	0.041	1.282	1.064–2.071
LATS1 expression (High vs. low)	0.0029	0.026	0.815	0.945–1.711

NA: not analyzed; NS: not significant.

### Ectopic expression of LATS1 inhibits cell growth and invasion

**Figure 2:**
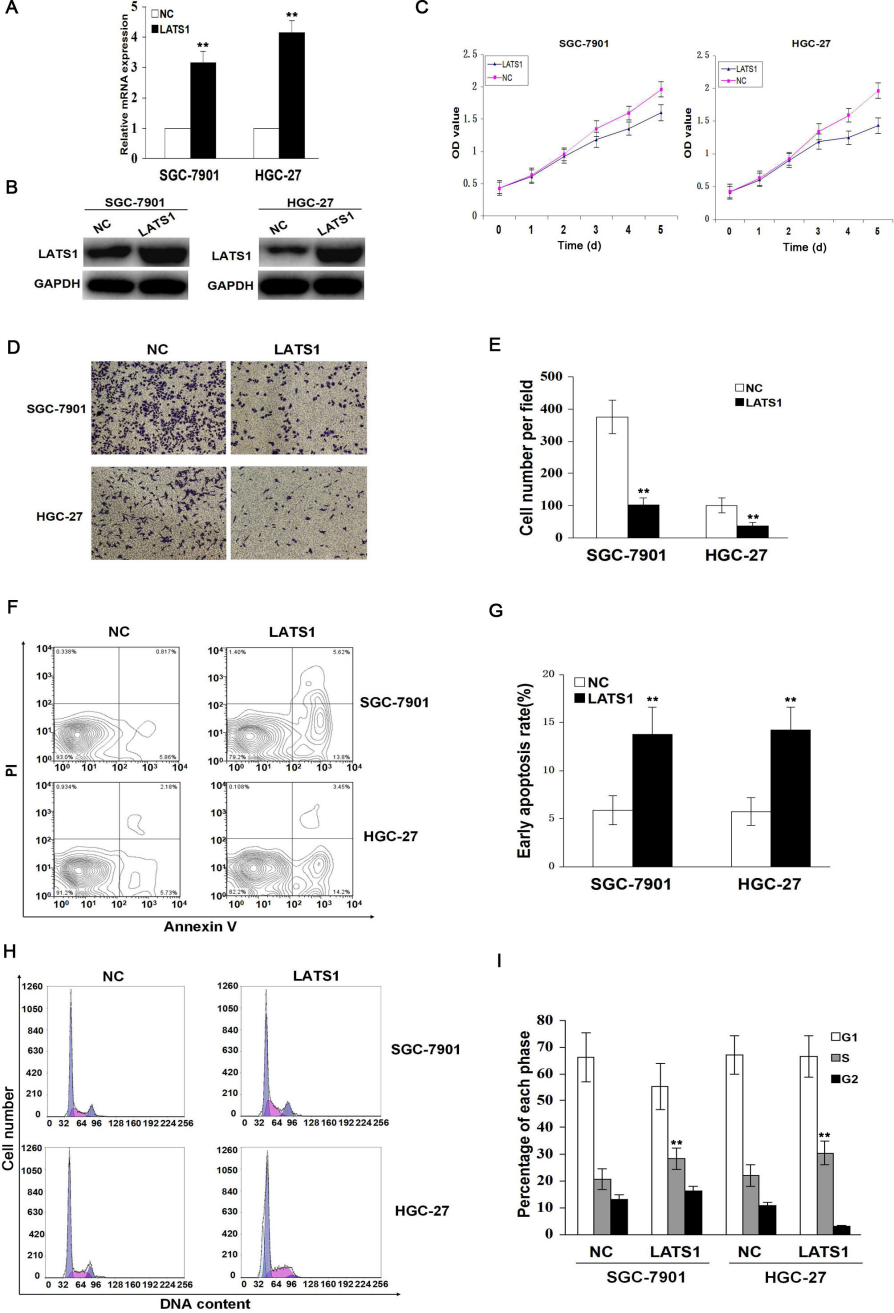
Overexpression of LATS1 inhibits cell growth and invasion (**A**) LATS1 mRNA and (**B**) protein expression were respectively detected by Real-time PCR and Western blotting in SGC-7901 and HGC-27 cells transfected with LATS1. (**C**) The effect of LATS1 overexpression on cell proliferative activity was estimated by MTT assay, indicating that overexpression of LATS1 reduced cell growth after LATS1 transfection for 72 h. (**D**) Representative micrographs of Transwell invasion assay were indicated in SGC-7901 and HGC-27 cells transfected with LATS1 (magnification, 200×). (**E**) Overexpression of LATS1 decreased cell invasive potential compared with the NC group. (**F**) Flow cytometry analysis of early apoptotic rate in SGC-7901 and HGC-27 cells after LAST1 transfection for 48 h. (**G**) Overexpression of LATS1 induced cell apoptosis compared with the NC group. (**H**) Flow cytometry analysis of cell cycle distribution in SGC-7901 and HGC-27 cells transfected with LATS1. (**I**) Overexpression of LATS1 induced cell cycle arrest at S phase compared with the NC group (**P < 0.01). The data were shown as the means ± SD of three independent experiments. **P < 0.01.

To find out whether LATS1 exerted a tumor suppressive function, LATS1 was stably overexpressed through a lentiviral vector system in SGC-7901 and HGC-27 cell lines with low endogenous LATS1 level. The LATS1 expression levels were identified by Real-time PCR (Figure 2A) and Western blotting analysis (Figure 2B). To observe whether LATS1 played a role in cell growth and invasion in GC cells, the cell proliferation and invasive potential were determined by MTT and Transwell assays, and cell apoptosis and cycle distribution were analyzed by flow cytometry. The results showed that overexpression of LATS1 significantly suppressed cell proliferation (Figure 2C, *P* < 0.01) and invasive capability (Figure 2D, 2E), and induced cell apoptosis (Figure 2F, 2G) and cell cycle arrest at S phase (Figure 2H, 2I) compared with the NC group in SGC-7901 and HGC-27 cells.

### LATS1 knockdown promotes cell growth and invasion

**Figure 3:**
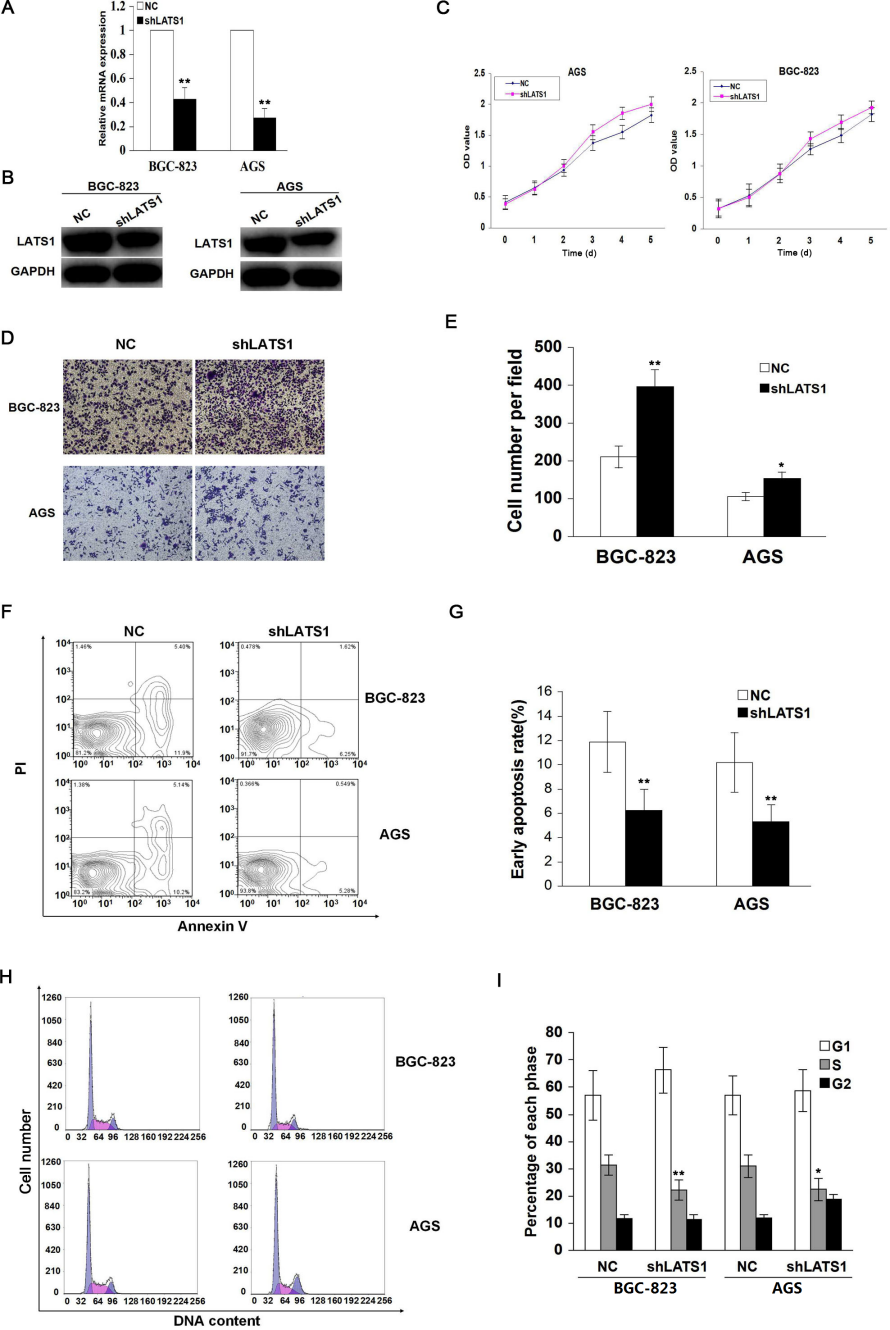
Knockdown of LATS1 promotes cell growth and invasion (**A**) LATS1 mRNA and (**B**) protein expression were respectively examined by Real-time PCR and Western blotting in BGC-823 and AGS cells transfected with shLATS1. (**C**) The effect of LATS1 knockdown on cell proliferative activity was evaluated by MTT assay, showing that LATS1 knockdown prompted cell growth after shLATS1 transfection for 72 h. (**D**) Representative micrographs of Transwell invasion assay were indicated in BGC-823 and AGS cells transfected with shLATS1 (magnification, 200×). (**E**) Knockdown of LATS1 enhanced cell invasive potential compared with the NC group. (**F**) Flow cytometry analysis of early apoptotic rate in BGC-823 and AGS cells after shLAST1 treatment for 48 h. (**G**) Knockdown of LATS1 cut down cell apoptosis compared with the NC group. (**H**) Flow cytometry analysis of cell cycle distribution in BGC-823 and AGS cells treated with shLATS1. (**I**) Knockdown of LATS1 decreased the cell cycle arrest at S phase compared with the NC group. The data were shown as the means ± SD of three independent experiments. *P < 0.05, **P < 0.01.

To determine whether LATS1 influenced the aggressive cellular phenotypes of GC cells, LATS1 gene was knocked down by a specific shRNA, and effective LATS1 silencing was decided by Real-time PCR (Figure 3A) and Western blotting analysis (Figure 3B) in AGS and BGC-823 cell lines with high endogenous LATS1 level. The results showed that knockdown of LATS1 significantly increased cell proliferation (Figure 3C) and invasive capability (Figure 3D, 3E), and decreased cell apoptosis (Figure 3F, 3G) and cycle arrest at S phase (Figure 3H, 3I) compared with the NC group.

### YAP signaling is required for LATS1-induced inhibition of cell growth and invasion

**Figure 4:**
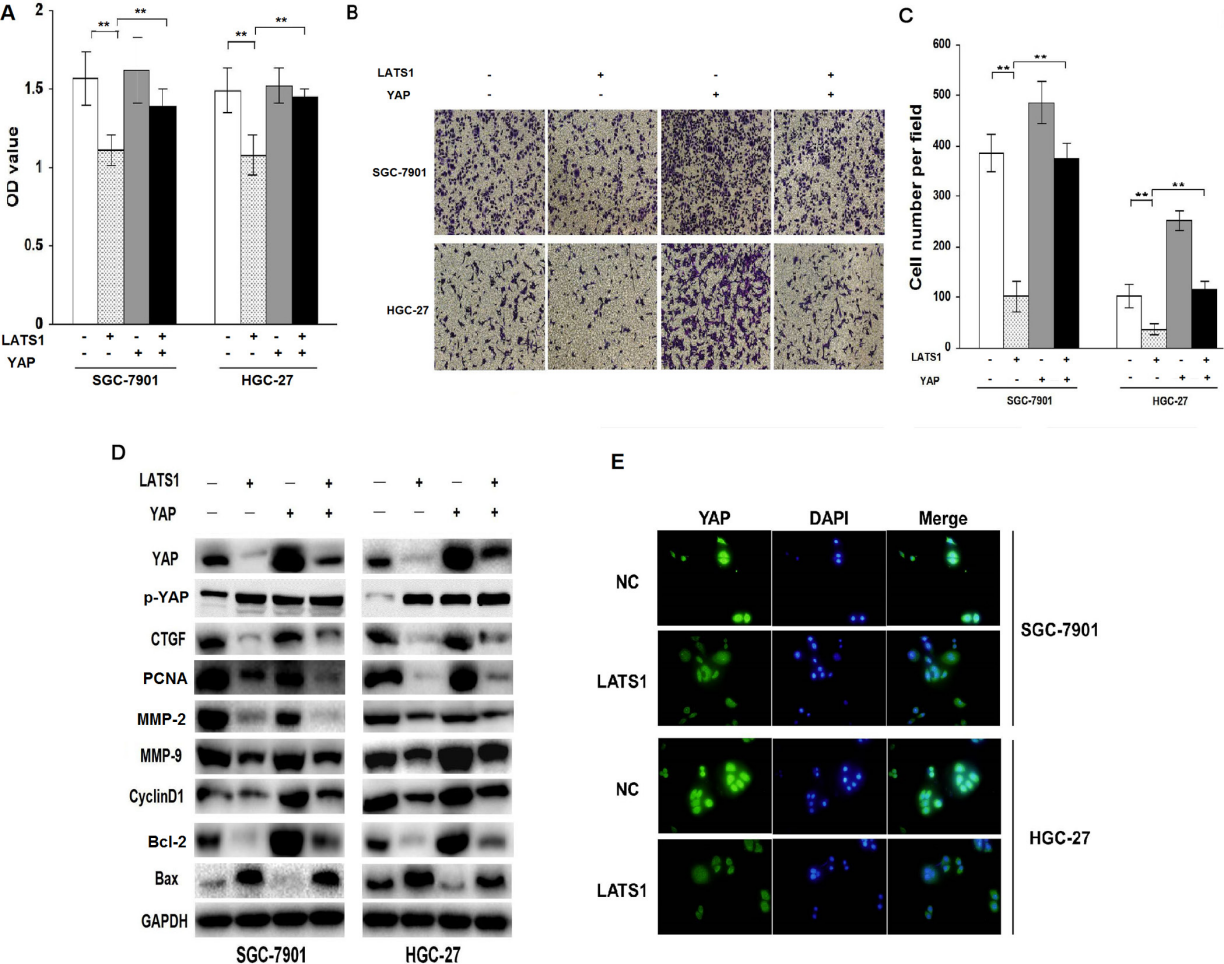
YAP signaling is required for LATS1-induced inhibition of cell growth and invasion (**A**) YAP-overexpressed vector was transfected into LATS1-pretreated GC cell lines (SGC-7901 and HGC-27). After transfection for 72 h, cell proliferative activity was assessed by MTT assay, indicating that YAP overexpression weakened the therapeutic effects of LATS1 on cell growth. (**B**, **C**) After YAP-overexpressed vector was transfected into LATS1-treated GC cell lines (SGC-7901 and HGC-27), cell invasive potential was determined by Transwell assay, demonstrating that YAP lowered the anti-invasion effect of LATS1 in GC cells. (**D**) Western blotting analysis of the YAP, p-YAP, CTGF, MMP-9, Bax and CyclinD1 expression in YAP-transfected SGC-7901 and HGC-27 cells treated with LATS1. (**E**) Immunofluorescence analysis of the location of YAP in SGC-7901 and HGC-27 cells transfected with LATS1. The data were shown as the means ± SD of three independent experiments. **P < 0.01.

To further explore the molecular mechanism and contribution of YAP signaling to LATS1-induced cell growth and invasion, lentivirus-mediated YAP-overexpressed vector was transfected into LATS1-overexpressed GC cell lines (SGC-7901 and HGC-27). After transfection for 72 h, cell proliferative activity (Figure 4A) and invasive potential (Figure 4B, 4C) was determined by MTT and Transwell assays, and YAP downstream factors related to cell growth and invasion were detected by Western blotting (Figure 4D). Moreover, immunofluorescence was performed to determine the distribution of YAP after transfection of LATS1 (Figure 4E). The results indicated that, on the one hand, YAP promoted cell growth and invasion but LATS1 reduced them not only by decreasing YAP, CTGF, PCNA, MMP-2, MMP-9, CyclinD1, Bcl-2 and increasing Bax and p-YAP expression, but also through inhibiting nuclear transfer of YAP. On the other hand, YAP overexpression attenuated the inhibitory effects of LATS1 transduction on cell growth, invasion and the expression of YAP, CTGF, PCNA, MMP-2, MMP-9, CyclinD1 and Bcl-2. Taken together, these findings suggested that LATS1 might inhibit the growth and invasion of GC cells through blockade of the YAP signaling.

### Overexpression of LATS1 inhibits tumor growth and metastatic potential *in vivo*

**Figure 5:**
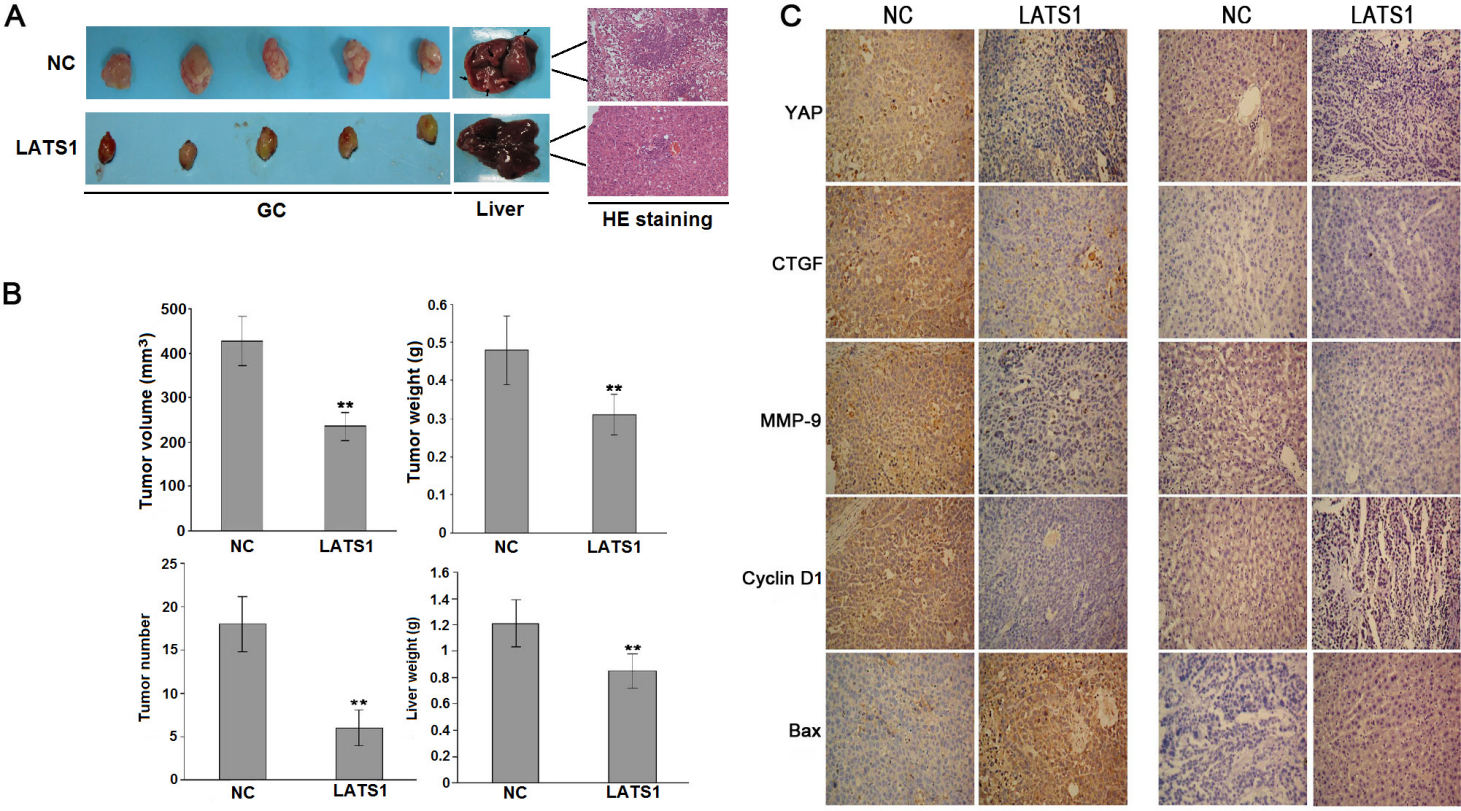
LATS1 suppressed the tumorigenicity and liver metastasis of SGC-7901 cells *in vivo* (**A**) Representative photographs of GC and metastatic liver tumors and HE staining of liver tissue derived from SGC-7901-LATS1 or SGC-7901-NC cells in SCID mice. (**B**) At the end of the experiment, the average volume and weight of gastric tumors in LATS1 group were significantly lower than those of the NC group. The number of metastatic liver tumor nodules and the average liver weight in LATS1 group were much lower than those of the NC group). (**C**) IHC analysis of the protein expression of YAP, CTGF, MMP-9, Bax and CyclinD1 in gastric tumors and metastatic liver tumors. The data were shown as the means ± SD of three independent experiments. **P < 0.01.

The *in vitro* experiments demonstrated the inhibitory effects of LATS1 overexpression on GC cells. Whether LATS1 exerting the same effect on the tumorigenicity and metastasis *in vivo* was further investigated. LATS1 and NC viruses-transfected SGC-7901 cells were used to establish SCID mice orthotopic implantation GC models. As indicated in Figure 5A, 5B, the average volume and weight of GC tissues in LATS1 group were significantly lower than those of the NC group, and the number of metastatic liver tumor nodules from GC and the average weight of the livers in LATS1 group were significantly lower than those of the NC group. In addition, IHC assay showed that LATS1 overexpression downregulated the expression of YAP, CTGF, MMP-9 and CyclinD1 and upregulated the expression of Bax in GC tissues and metastatic liver tumors (Figure 5C).

## DISCUSSION

LATS1 as a central player of the Hippo pathway is involved in regulation of the organ size and cell proliferation. Previous studies have shown that the expression of LATS1 is downregulated in malignant mesothelioma [33], glioma [34], NSCLC [35] and ovarian tumors [36] and represents an independent prognostic indicator for the survival and prognosis of patients with glioma and ovarian cancer [34–36]. Deficiency of LATS1 directly brings about the tumor formation of soft-tissue sarcomas and ovarian tumors in mice [37] and facilitates the progression of glioma [34]. However, little is reported regarding its expression and clinical significance of LATS1 in GC. To search the possible biological functions of LATS1 in GC, we detected the expression of LATS1 in GC tissues and cell lines at the protein level, indicating that LATS1 was markedly downregulated in GC tissues and invasive SGC-7901 and HGC-27 cell lines. Importantly, decreased expression of LATS1 was correlated with the lymphatic node metastasis, and GC patients with a low level of LATS1 expression indicated poor OS and RFS, suggesting that LATS1 might serve as an independent factor for OS and RFS in GC patients.

Accumulating evidence has shown that LATS1 as a new resident governor of cellular homeostasis possesses multiple biological functions through the regulation of cell proliferation, cell apoptosis and cell migration and invasion [5]. LATS1 inhibits cell proliferation and vascular invasion and induces cell apoptosis and cycle arrest [8, 38, 39], indicating LATS1 as a tumor suppressor in human cancer [15]. Consistent with these results, our present studies supported that LATS1 acted as a tumor suppressor in GC. To further clarify the biological functions of LATS1 in GC, we evaluated the effect of LATS1 overexpression on GC cell proliferation and invasive potential by MTT and Transwell assays. As a result, the overexpression of LAST1 suppressed cell proliferation and invasion in SGC-7901 and HGC-27 cell lines with low endogenous LATS1 level, whereas LATS1 silencing promoted cell proliferation and invasion in AGS and BGC-823 cells presenting high endogenous LATS1 level. Flow cytometry indicated that induction of apoptosis and cell cycle arrest by LATS1 may contribute to the tumor suppressive function. S phase represents the period of DNA synthesis and our research found LATS1 overexpression blocked GC cell cycle in S phase, as a result of which, the GC proliferation and invasion were inhibited by LATS1. In addition, a SCID mice model *in vivo* revealed that the overexpression of LATS1 significantly reduced the capability of GC cells to induce tumorigenesis and liver metastases.

**Figure 6:**
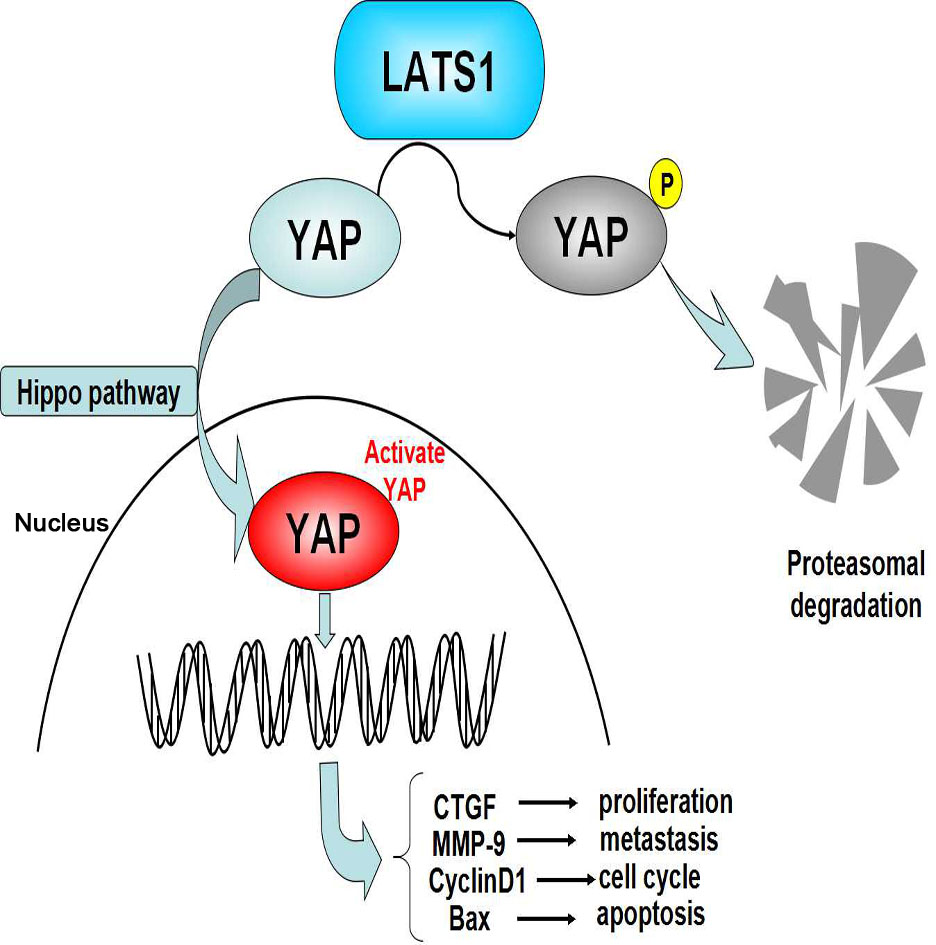
Loss of LATS1 might promote the tumorigenesis of GC via upregulation of the YAP signaling YAP is required for LATS1-induced inhibition of GC cell proliferation and invasion through upregulation of CTGF, MMP-9 and CyclinD1 expression and downregulation of Bax expression.

YAP as a downstream effector of the Hippo pathway acts an oncogenic role in cellular proliferation and apoptosis [40]. It is overexpressed in a series of tumorigenetic models and human cancers and promotes the progression of malignant tumors [41, 42]. Moreover, previous studies have shown that LATS1 negatively regulates YAP-oncogenic function via phosphorylation of YAP in Drosophila [43], mammalian cells [44] and non-small-cell lung cancer (NSCLC) [45]. But, whether YAP is involved in the regulation of LATS1 in GC cells is not comprehensively understood. In this study, we found that YAP overexpression promoted cell growth and invasion and its downregulation was required for the LATS1-induced inhibition of cell growth and invasion in GC cells. In addition, YAP has been confirmed to be involved in the regulation of downstream factors including CTGF [46], Bax [47], MMP-9 [32] and CyclinD1 [48], which are related to tumor proliferation, metastasis and angiogenesis in NSCLC and GC cells. Furthermore, we found that LATS1 overexpression suppressed cell growth and invasion through downregulation of YAP, CTGF, PCNA, MMP-2, MMP-9, Bcl-2 and CyclinD1 expression and upregulation of p-YAP and Bax expression. Decreasing YAP expression and increasing p-YAP expression were in accordance with our study showing that LATS1 inhibited nuclear transfer of YAP. These findings suggested that LATS1 might function as a tumor suppressor in GC cells via inhibition of the YAP signaling (Figure 6).

Our present findings provide the demonstration that LATS1 expression is frequently decreased in GC tissues, and loss of LATS1 expression is associated with tumor invasion, poor prognosis and recurrence in GC patients. LATS1 overexpression inhibits cell growth and invasion *in vitro* and *in vivo* via inhibition of the YAP signaling, suggesting that LATS1 may be an important therapeutic target for the treatment of GC.

## MATERIALS AND METHODS

### Materials

Human GC tissues and the corresponding adjacent non-tumor tissues (ANTT) were collected from Shanghai Jiao Tong University affiliated Shanghai Sixth People's Hospital. GC tissue microarray was made by Shanghai Outdo Biotech CO. LTD (Shanghai, PR, China). GC cell lines (SGC-7901, BGC-823, HGC-27, AGS) and gastric epithelial cells (GES-1) used in these experiments were from Laboratory of Gastroenterology of Shanghai Sixth People's Hospital. Lentivirus-mediated YAP overexpression vector, lentivirus-mediated LATS1 shRNA vector (shLATS1), negative control vector (NC), and virion-packaging elements were purchased from Genechem (Shanghai, PR, China); All antibodies used in this study were purchased from Cell Signaling Technologies (Beverly, MA, USA). Six-week-old female SCID mice were purchased from Shanghai Laboratory Animal Center of Chinese Academy Sciences (SPF, NO.SCXK033) (Shanghai, PR, China).

### Drugs and reagents

Dulbecco's Modified Eagle medium (DMEM) and fetal bovine serum (FBS) were from Thermo Fisher Scientific Inc (Waltham, MA, USA); 3-(4,5)-dimethylthiahiazo (-z-yl)-3,5- di-phenytetrazoliumromide (MTT) was from Dingguo biology (Shanghai, China); TRIzol Reagent and Lipofectamine 2000 were from Invitrogen (Carlsbad, CA, USA); M-MLV Reverse Transcriptase was from Promega (Madison, WI, USA); SYBR Green Master Mixture was from Takara (Otsu, Japan); Cell cycle analysis kit, apoptosis kit (Propidium Iodide (PI), RNase A, Annexin V-FITC) and all antibodies for immunohistochemistry, western blot and immunofluorescence were from KeyGEN biology (Nanjing, China). ECL-PLUS/Kit was from GE Healthcare (Piscataway, NJ, USA).

### Clinical samples and data

Tumor tissues were collected from biopsy samples undergoing resection of the primary GC in a total of 89 consecutive cases admitted in our hospital from January 2005 to September 2013. The baseline characteristics of the patients before neo-adjuvant chemotherapy were summarized. Follow-up studies included physical examination, laboratory analysis, and computed tomography if necessary. Overall survival (OS) was defined as the interval between the dates of surgery and death. Recurrence free survival (RFS) was defined as the interval between the dates of surgery and recurrence. The study was approved by Medical Ethics Committee of Shanghai Jiao Tong University and written informed consent was obtained from the patients or their parents before sample collection. Two pathologists respectively checked all the cases.

### Immunohistochemical staining

Anti-LATS1 antibody was used for immunohistochemical (IHC) detection of the expression of LATS1 protein in tissue microarray, whose sections were processed for IHC analysis as follows: IHC examinations were carried out on 3 mm thick sections. For anti-LATS1 IHC, unmasking was performed with 10 mM sodium citrate buffer, pH 6.0, at 90°C for 30 min. Sections were incubated in 0.03% hydrogen peroxide for 10 min at room temperature, to remove endogenous peroxidase activity, and then in blocking serum (0.04% bovine serum albumin, A2153, Sigma-Aldrich, Shanghai, China and 0.5% normal goat serum X0907, Dako Corporation, Carpinteria, CA, USA, in PBS) for 30 min at room temperature. Anti-LATS1 antibody was used at a dilution of 1:200. The antibody was incubated overnight at 4°C. Sections were then washed three times for 5 min in PBS. Non-specific staining was blocked with 0.5% casein and 5% normal serum for 30 min at room temperature. Finally, staining was developed using diaminobenzidine substrate, and sections were counterstained with hematoxylin. Normal serum or PBS was used to replace LATS1 antibody in negative control.

The expression level of LATS1 was semiquantitatively counted as the total immunostaining scores, calculated as the product of a proportion score and an intensity score. The proportion score reflected the fraction of positive staining cells (0, none; 1, ≤ 10%; 2, 10% to ≥ 25%; 3, > 25% to 50%; 4, > 50%), and the intensity score represented the staining intensity (0, no staining; 1, weak; 2, intermediate; 3, strong). Finally, a total score was given ranging from 0 to 12. Based on the analysis in advance, LATS1 expression was categorized into negative (score 0), weak (score 1–3), intermediate (score 4–6), and strong (score 7–12). Score < 4 was defined as low expression while score ≥ 4 was considered as high expression. The scoring was independently assessed by two pathologists.

### Construction of vectors

The full-length CDS of human YAP was amplified from the cDNA from HEK293T cells using the following primers: forward: 5′-CCTGATGGATGGGAACAAGC-3′; reverse 5′- GCACTCTGACTGATTCTCTGG-3′. The PCR product was purified using a PCR purification kit and cloned into the PGC-LV neo lentivirus vector that was driven by the U6 promoter and carried the transgene for green fluorescent protein. The inserted sequences of the vectors were confirmed by sequencing. To silence the expression of LATS1, short hairpin RNA (shRNA) sequence targeting LATS1 gene (clone: ATCCTCGACGAGAGCAGA) were purchased from Genechem (Shanghai, PR, China).

### Cell culture and lentiviral infections

GC cells were cultured in DMEM medium supplemented with 10% heat-inactivated FBS, 100U/ml of penicillin, and 100 μg/ml of streptomycin. Cells in this medium were placed in a humidified atmosphere containing 5% CO_2_ at 37°C. On the day of transduction, GC cells were replated at 5 × 10^4^ cells/well in 24-well plates containing serum-free growth medium with polybrene (5 mg/ml). When reached 50% confluence, cells were transfected with recombinant experimental virus or control virus at the optimal MOI (multiplicity of infection) of 50, and cultured at 37°C and 5% CO2 for 4 h. Then supernatant was discarded and serum containing growth medium was added. Positive and stable transfectants were selected and expanded for further study. The LATS1 shRNA lentivirus-infected clone and the negative control-infected cells were set as shLATS1 group and NC group.

### Quantitative Real-time PCR

To quantitatively confirm the mRNA expression levels of LATS1 in GC cell lines, real-time PCR was performed. Total RNA was extracted from each clone using TRIzol according to the manufacturer's protocol. Reverse transcription was carried out using M-MLV and cDNA amplification was performed using the SYBR Green Master Mix kit according to the manufacturer's guidelines. The LATS1 gene was amplified using a specific oligonucleotide primer: sense 5′-GTTAAGGGGAGAGCCAGGTCCTT-3′ and antisense 5′-TCAAGGAAGTCCCCAGG ACTGT-3′. Human glyceraldehyde-3-phosphate dehydrogenase (GAPDH) gene was used as an endogenous control. Data were analyzed using the comparative Ct method (2^−ΔΔCt^). Three separate experiments were performed for each clone.

### Western blot analysis

GC cell lines were harvested and extracted using lysis buffer (Tris-HCl, SDS, Mercaptoethanol, Glycerol). Cell extracts were boiled for 5 min in loading buffer and then equal amount of cell extracts were separated on 15% SDS-PAGE gels. Separated protein bands were transferred into polyvinylidene fluoride (PVDF) membranes and the membranes were blocked in 5% skim milk powder. The primary antibodies against LATS1, YAP, p-YAP, CTGF, PCNA, MMP-2, MMP-9, Bcl-2, Bax and CyclinD1 were diluted according to the instructions of antibodies and incubated overnight at 4°C. Then, horseradish peroxidase-linked secondary antibodies were added at a dilution ratio of 1:1000, and incubated at room temperature for 2 h. The membranes were washed with PBS for three times and the immunoreactive bands were visualized using ECL-PLUS/Kit according to the kit's instruction. The relative protein level in different groups was normalized to GAPDH concentration. Three separate experiments were performed for each clone.

### Cell viability assay

Cell proliferation was analyzed using the MTT assay. Briefly, GC cells transfected with Lv-LATS1 were incubated in 96-well-plates at a density of 1 × 10^5^ cells per well with DMEM medium supplemented with 10% FBS. Cells were treated with 20 μl of MTT dye at 0, 24 h, 48 h, 72 h, 96 h, 120 h and subsequently incubated with 150 μl of DMSO for 5 min. The color reaction was measured at 570 nm using an Enzyme Immunoassay Analyzer (Bio-Rad, Hercules, CA). The proliferation activity was calculated for each clone.

### Transwell invasion assay

Transwell filters were coated with matrigel (3.9 μg/μl, 60–80 μl) on the upper surface of a polycarbonic membrane (diameter 6.5 mm, pore size 8 μm). After incubating at 37°C for 30 min, the matrigel solidified and served as the extracellular matrix for analysis of tumor cell invasion. Harvested cells (1 × 10^5^) in 100 μl of serum free DMEM were added into the upper compartment of the chamber. A total of 200 μl conditioned medium derived from NIH3T3 cells was used as a source of chemoattractant, and was placed in the bottom compartment of the chamber. After 24 h incubation at 37°C with 5% CO_2_, the medium was removed from the upper chamber. The non-invaded cells on the upper side of the chamber were scraped off with a cotton swab. The cells that had migrated from the matrigel into the pores of the inserted filter were fixed with 100% methanol, stained with Hematoxylin, and mounted and dried at 80°C for 30 min. The number of cells invading through the matrigel was counted in three randomly selected visual fields from the central and peripheral portion of the filter using an inverted microscope (200 × magnification). Each assay was repeated three times.

### Flow-cytometric analysis

To detect cell apoptosis, GC cells were trypsinized, washed with cold PBS and resuspended in binding buffer according to the instruction of the apoptosis kit. FITC-AnnexinV and PI were added to the fixed cells for 20 min in darkness at room temperature. Then, Annexin V binding buffer was added to the mixture before the fluorescence was measured on FAC sort flow cytometer. The cell apoptosis was analyzed using the Cell Quest software (Becton Dickinson, USA). Three separate experiments were performed for each clone.

After PBS washing, the fixed cells were stained with PI in the presence of RNase A for 30 min at room temperature in darkness. Each sample was filtered through a 50 μm nylon filter to obtain single-cell suspension. The samples were then analyzed on FACsort flow cytometer (Becton Dickinson, Mountain View, CA, USA). ModFit 3.0 software (Verity Software House, Topsham, ME, USA) was used for cell cycle analysis. Three separate experiments were performed for each clone.

### Immunofluorescence assays

Cells were fixed in methanol for 15 min, permeabilized in 0.2% Triton X-100 for 20 min and blocked in PBS containing 5% BSA overnight at 4°C. The cells were incubated with YAP (1:200) antibodies at 4°C overnight and washed by PBS for three times. Then the cells were treated with Alexa Fluor 488 goat anti-rabbit IgG (1:800) for 1 h in a lucifugal chamber. The samples were washed with PBS for three times followed by treatment with DAPI Fluoromount G. Images were acquired using an Olympus BX51 microscope coupled with an Olympus DP70 digital camera.

### *In vivo* tumorigenesis and metastasis assay

SGC-7901 GC cell line was subcutaneously injected and maintained by passage in the hypodermis of nude mice. Animal models were made using orthotopic implantation of histological intact GC tissue. Tumor tissues were resected aseptically. Necrotic tissues were cut and the remaining healthy tumor tissues were scissor minced into pieces (about 2 mm × 2 mm in diameter) in Hank's balanced salt solution. Each tumor piece was weighed and adjusted to 50 mg. Mice were anesthetized with 4.3% trichloraldehyde hydrate. An incision was made through the left upper abdominal pararectal line. Then the peritoneal cavity was carefully exposed and a part of the serosa membrane in the middle of the greater curvature of the stomach was mechanically injured using scissors. A tumor piece was fixed on each injured site of the serosal surface. The stomach was returned to the peritoneal cavity, and the abdominal wall and skin were closed. After 7 days, mice were randomly separated into 2 groups with 5 mice per group. After three weeks, mice were sacrificed by cervical dislocation. The tumor volume was measured with a caliper, using the formula volume = (length × width^2^)/2. Samples of the tumor were immediately frozen in liquid nitrogen for later use.

### Statistical analysis

SPSS 20.0 was used for the statistical analysis. All of the values were recorded as the mean ± SEM from at least 3 independent experiments. Kruskal-Wallis H test and Chi-square test were used to analyze the expression rate in all groups. One-way analysis of variance (ANOVA) was used to analyze the differences between groups. The LSD method of multiple comparisons was used when the probability for ANOVA was statistically significant. Statistical significance was set at *P* < 0.05.

## References

[1] Torre LA, Bray F, Siegel RL, Ferlay J, Lortet-Tieulent J, Jemal A (2015). Global cancer statistics, 2012. CA Cancer J Clin.

[2] Yamamoto M, Rashid OM, Wong J (2015). Surgical management of gastric cancer: the East vs. West perspective. J Gastrointest Oncol.

[3] Nishida T, Doi T (2014). Improving prognosis after surgery for gastric cancer. Lancet Oncol.

[4] Bornschein J, Weigt J, Selgrad M, Malfertheiner P (2009). Molecular aspects in the diagnosis of gastric cancer. Expert Opin Med Diagn.

[5] Visser S, Yang X (2010). LATS tumor suppressor: a new governor of cellular homeostasis. Cell Cycle.

[6] Justice RW, Zilian O, Woods DF, Noll M, Bryant PJ (1995). The Drosophila tumor suppressor gene warts encodes a homolog of human myotonic dystrophy kinase and is required for the control of cell shape and proliferation. Genes Dev.

[7] Xu T, Wang W, Zhang S, Stewart RA, Yu W (1995). Identifying tumor suppressors in genetic mosaics: the Drosophila lats gene encodes a putative protein kinase. Development.

[8] Xia H, Qi H, Li Y, Pei J, Barton J, Blackstad M, Xu T, Tao W (2002). LATS1 tumor suppressor regulates G2/M transition and apoptosis. Oncogene.

[9] Zeng Q, Hong W (2008). The emerging role of the hippo pathway in cell contact inhibition, organ size control, and cancer development in mammals. Cancer Cell.

[10] Zhou Y, Tao F, Cheng Y, Xu F, Yao F, Feng D, Miao L, Xiao W, Ling B (2014). Up-regulation of ITCH is associated with down-regulation of LATS1 during tumorigenesis and progression ofcervical squamous cell carcinoma. Clin Invest Med.

[11] Morinaga N, Shitara Y, Yanagita Y, Koida T, Kimura M, Asao T, Kimijima I, Takenoshita S, Hirota T, Saya H, Kuwano H (2000). Molecular analysis of the h-warts/LATS1 gene in human breast cancer. Int J Oncol.

[12] Li H, Wolfe A, Septer S, Edwards G, Zhong X, Abdulkarim AB, Ranganathan S, Apte U (2012). Deregulation of Hippo kinase signalling in human hepatic malignancies. Liver Int.

[13] Jiang Z, Li X, Hu J, Zhou W, Jiang Y, Li G, Lu D (2006). Promoter hypermethylation-mediated down-regulation of LATS1 and LATS2 in human astrocytoma. Neurosci Res.

[14] Yu T, Bachman J, Lai ZC (2013). Evidence for a tumor suppressor role for the large tumor suppressor genes LATS1 and LATS2 in human cancer. Genetics.

[15] Yu T, Bachman J, Lai ZC (2015). Mutation analysis of large tumor suppressor LATS1 and LATS2 supports a tumor suppressor role inhuman cancer. Protein Cell.

[16] Saadeldin MK, Shawer H, Mostafa A, Kassem NM, Amleh A, Siam R (2015). New genetic variants of LATS1 detected in urinary bladder and colon cancer. Front Genet.

[17] Pan D (2007). Hippo signaling in organ size control. Genes Dev.

[18] Zender L, Spector MS, Xue W, Flemming P, Cordon-Cardo C, Silke J, Fan ST, Luk JM, Wigler M, Hannon GJ, Mu D, Lucito R, Powers S (2006). Identification and validation of oncogenes in liver cancer using an integrative oncogenomic approach. Cell.

[19] Zhao B, Wei X, Li W, Udan RS, Yang Q, Kim J, Xie J, Ikenoue T, Yu J, Li L, Zheng P, Ye K, Chinnaiyan A (2007). Inactivation of YAP oncoprotein by the Hippo pathway is involved in cell contact inhibition and tissue growth control. Genes Dev.

[20] Perra A, Kowalik MA, Ghiso E, Ledda-Columbano GM, Di Tommaso L, Angioni MM, Raschioni C, Testore E, Roncalli M, Giordano S, Columbano A (2014). YAP activation is an early event and a potential therapeutic target in liver cancer development. J Hepatol.

[21] Wang Y, Dong Q, Zhang Q, Li Z, Wang E, Qiu X (2010). Overexpression of yes-associated protein contributes to progression and poor prognosis of non-small cell lung cancer. Cancer Sci.

[22] Kim SK, Jung WH, Koo JS (2014). Yes-associated protein (YAP) is differentially expressed in tumor and stroma according to the molecular subtype of breast cancer. Int J Clin Exp Pathol.

[23] Yuan H, Liu H, Liu Z, Zhu D, Amos CI, Fang S, Lee JE, Wei Q (2015). Genetic variants in Hippo pathway genes YAP1, TEAD1 and TEAD4 are associated with melanoma-specific survival. Int J Cancer.

[24] Fernandez-L A, Northcott PA, Dalton J, Fraga C, Ellison D, Angers S, Taylor MD, Kenney AM (2009). YAP1 is amplified and up-regulated in hedgehog-associated medulloblastomas and mediates Sonic hedgehog-driven neural precursor proliferation. Genes Dev.

[25] Steinhardt AA, Gayyed MF, Klein AP, Dong J, Maitra A, Pan D, Montgomery EA, Anders RA (2008). Expression of Yes-associated protein in common solid tumors. Hum Pathol.

[26] Ye XY, Luo QQ, Xu YH, Tang NW, Niu XM, Li ZM, Shen SP, Lu S, Chen ZW (2015). J Cell Mol Med. 17-AAG suppresses growth and invasion of lung adenocarcinoma cells via regulation of the LATS1/YAP pathway.

[27] Tufail R, Jorda M, Zhao W, Reis I, Nawaz Z (2012). Loss of Yes-associated protein (YAP) expression is associated with estrogen and progesterone receptors negativity in invasive breast carcinomas. Breast Cancer Res Treat.

[28] Huntoon CJ, Nye MD, Geng L, Peterson KL, Flatten KS, Haluska P, Kaufmann SH, Karnitz LM (2010). Heat shock protein 90 inhibition depletes LATS1 and LATS2, two regulators of the mammalian hippo tumorsuppressor pathway. Cancer Res.

[29] Lin XY, Zhang XP, Wu JH, Qiu XS, Wang EH (2014). Expression of LATS1 contributes to good prognosis and can negatively regulate YAP oncoprotein in non-small-cell lung cancer. Tumour Biol.

[30] Wang C, Zhu ZM, Liu CL, He XJ, Zhang HY, Dong JH (2015). Knockdown of yes-associated protein inhibits proliferation and downregulates large tumorsuppressor 1 expression in MHCC97H human hepatocellular carcinoma cells. Mol Med Rep.

[31] Xu ZP, Zhu JS, Zhang Q, Wang XY (2011). A breakdown of the Hippo pathway in gastric cancer. Hepatogastroenterology.

[32] Zhang J, Xu ZP, Yang YC, Zhu JS, Zhou Z, Chen WX (2012). Expression of Yes-associated protein in gastric adenocarcinoma and inhibitory effects of its knockdown on gastric cancer cell proliferation and metastasis. Int J Immunopathol Pharmacol.

[33] Miyanaga A, Masuda M, Tsuta K, Kawasaki K, Nakamura Y, Sakuma T, Asamura H, Gemma A, Yamada T (2015). Hippo pathway gene mutations in malignant mesothelioma: revealed by RNA and targeted exon sequencing. J Thorac Oncol.

[34] Ji T, Liu D, Shao W, Yang W, Wu H, Bian X (2012). Decreased expression of LATS1 is correlated with the progression and prognosis of glioma. J Exp Clin Cancer Res.

[35] Lin XY, Zhang XP, Wu JH, Qiu XS, Wang EH (2014). Expression of LATS1 contributes to good prognosis and can negatively regulate YAP oncoprotein in non-small-cell lung cancer. Tumour Biol.

[36] Xu B, Sun D, Wang Z, Weng H, Wu D, Zhang X, Zhou Y, Hu W (2015). Expression of LATS family proteins in ovarian tumors and its significance. Hum Pathol.

[37] St John MA, Tao W, Fei X, Fukumoto R, Carcangiu ML, Brownstein DG, Parlow AF, McGrath J, Xu T (1999). Mice deficient of Lats1 develop soft-tissue sarcomas, ovarian tumours and pituitary dysfunction. Nat Genet.

[38] Sharif GM, Schmidt MO, Yi C, Hu Z, Haddad BR, Glasgow E, Riegel AT, Wellstein A (2015). Cell growth density modulates cancer cell vascular invasion via Hippo pathway activity and CXCR2 signaling. Oncogene.

[39] Yang X, Li DM, Chen W, Xu T (2001). Human homologue of Drosophila lats, LATS1, negatively regulate growth by inducing G(2)/M arrest or apoptosis. Oncogene.

[40] Overholtzer M, Zhang J, Smolen GA, Muir B, Li W, Sgroi DC, Deng CX, Brugge JS, Haber DA (2006). Transforming properties of YAP, a candidate oncogene on the chromosome 11q22 amplicon. Proc Natl Acad Sci U S A.

[41] Moroishi T, Hansen CG, Guan KL (2015). The emerging roles of YAP and TAZ in cancer. Nat Rev Cancer.

[42] Barron DA, Kagey JD (2014). The role of the Hippo pathway in human disease and tumorigenesis. Clin Transl Med.

[43] Hao Y, Chun A, Cheung K, Rashidi B, Yang X (2008). Tumor suppressor LATS1 is a negative regulator of oncogene YAP. J Biol Chem.

[44] Zhang J, Smolen GA, Haber DA (2008). Negative regulation of YAP by LATS1 underscores evolutionary conservation of the Drosophila Hippo pathway. Cancer Res.

[45] Lin XY, Zhang XP, Wu JH, Qiu XS, Wang EH (2014). Expression of LATS1 contributes to good prognosis and can negatively regulate YAP oncoprotein in non-small-cell lung cancer. Tumour Biol.

[46] You B, Yang YL, Xu Z, Dai Y, Liu S, Mao JH, Tetsu O, Li H, Jablons DM, You L (2015). Inhibition of ERK1/2 down-regulates the Hippo/YAP signaling pathway in human NSCLC cells. Oncotarget.

[47] Zhang H, Wu S, Xing D (2011). YAP accelerates Aβ(25–35)-induced apoptosis through upregulation of Bax expression by interaction with p73. Apoptosis.

[48] Zhang YH, Li B, Shen L, Shen Y, Chen XD (2013). The role and clinical significance of YES-associated protein 1 in human osteosarcoma. Int J Immunopathol Pharmacol.

